# The Retrospective Address Delivered at the Tenth Anniversary Meeting of the Provincial Medical and Surgical Association, Held at Exeter, Aug. 3d and 4th, 1842

**Published:** 1843-01

**Authors:** 


Art. VI.—*The Retrospective Address delivered at the Tenth Anniversary Meeting of the Provincial Medical and Surgical Association, held at Exeter, Aug. 3d and 4th,* 1842. By James Black, m.d. &c. —*Worcester,* 1842. 8vo, pp. 146.

This is a document full of interesting and valuable matter, and, therefore, well worthy the attention of the profession. We think it both too long and too minute for a spoken address; but it is on this very account of more value to the reader. If the Provincial Association did nothing more than give occasion to these annual retrospective surveys of the medical sciences it would deserve all the encouragement it receives.

